# Parent Participation in a Support Group for Families with Transgender and Gender-Nonconforming Children: “Being in the Company of Others Who Do Not Question the Reality of Our Experience”

**DOI:** 10.1089/trgh.2018.0018

**Published:** 2019-08-12

**Authors:** Amy Hillier, Elisabeth Torg

**Affiliations:** ^1^ School of Social Policy & Practice, University of Pennsylvania, Philadelphia, Pennsylvania.; ^2^ Friends Council on Education, Philadelphia, Pennsylvania.

**Keywords:** emotional support, gender nonconforming children, parents, shared language, support group, transgender children

## Abstract

**Purpose:** Parent support is considered crucial for the health of transgender and gender-nonconforming (trans/GNC) children, yet little research has focused on how to support parents and caregivers. This study considered the experience of participation in a support group for parents of transgender children on families.

**Methods:** Online surveys were conducted with parents whose children were receiving clinical care at a gender specialty clinic and who participated at least once in a monthly support group. Close-ended questions addressed the importance of participation and open-ended questions addressed the specific ways it was helpful, how it impacted them and their trans/GNC child, and if they faced barriers to participating.

**Results:** The majority of the 48 participants (77.1%) identified as female. The mean age of their trans/GNC child was 13.9 years (standard deviation 5.1, range 5–22 years); just over half (*n*=25) of their trans/GNC children identified as male. Participants overwhelmingly reported positive experiences with the support group, with 72.9% reporting that the group was either important or critically important to them and 66.7% reporting that it was important or critically important to their trans/GNC child. Perceived benefits included the opportunity to learn about legal, medical, and school issues and receive emotional support.

**Conclusion:** Support groups provide an important complement to specialized clinical care for families with trans/GNC children. Logistical challenges, lack of age peers, and lack of people of color all served as limitations of the group. Further research is needed to understand the experiences of fathers and to determine if the support group model would be effective with racially/ethnically and economically diverse populations.

## Introduction

Parents play a critical role in the health and well-being of transgender and gender-nonconforming (trans/GNC) children and youth, according to health care providers, advocates, and researchers.^1,2^ Clinicians and researchers, alike, regularly measure the level of parent acceptance, leading to the development of a variety of measures, including those completed by parents and their trans/GNC children.^3,4^ Research on family support of transgender children has shown that support from parents may protect against depression and anxiety and is associated with high life satisfaction among trans/GNC children and youth.^5,6^ From a family systems theory framework, the needs of parents must be addressed as well as the needs of their trans/GNC children because a child's gender transition requires families to “renegotiate identity and relationships” throughout the family system.^7,8^ Research that has focused on parents of trans/GNC children has identified their need for both professional and peer support to address their relevant medical, emotional, political, and financial questions and needs.^9,10^

Increasingly, the medical and public health literature are beginning to address the specific medical needs of trans/GNC children,^1,11–12^ but there is little research about the broader needs of their families, including the need for peer support among parents. Peer support in the context of a family member's mental, behavioral, and physical health condition often takes the form of self-help or support groups.^13–16^ Ainbinder et al. identified multiple benefits of support group participation, in general, for parents: (1) perceived sameness, meaning connection and “no judgments”; (2) comparable situations in other families that allowed for learning skills and gathering information; and (3) mutuality of support through equitable or peer relationships.^17^ In describing participation in a support group for parents of children with attention-deficit hyperactivity disorder (ADHD), Frigerio and Montali^18^ point to the production of “a shared narrative” that “served to absolve parents of guilt,” helped parents to distance themselves from the “blaming social context,” and preserved their identities as “good parents.” Passa and Giovazolis found that support group participation by parents led to “decrease of anxiety, the adoption of effective coping strategies, and the improvement of family dynamics” for families with a child suffering from substance abuse.”^19^

We know little about the specific experiences of parents of trans/GNC children with support groups. Menvielle and Tuerk, a psychiatrist and psychoanalyst, respectively, published a study based on lessons they learned from leading a 90-min monthly support group for parents of gender-nonconforming boys older than 3.^20^ Their results highlight the specific concerns parents voiced during the group about their child's gender confusion, playmates, bullying and sexuality and their own grieving, embarrassment, and shame, and uncertainty about disclosing their child's gender identity. One more recent study points to the importance of the Internet for obtaining information and social support for parents of trans/GNC children,^21^ but no published studies other than Menvielle and Tuerk have considered the value of in-person support groups for parents of trans/GNC children.

Led by two parents of transgender children, this study presents survey results from parents of trans/GNC children and youth who have participated in a monthly support group. Through a largely qualitative analysis, we seek to address the following research questions: (1) what benefits do parents perceive for themselves, their trans/GNC children, and other family members from participating in a support group? and (2) what are the limitations of and barriers to participation?

## Methods

### Study setting and recruitment

Both authors participate in a monthly support group for families with transgender/GNC children. Only families receiving clinical care from the Gender and Sexuality Development Clinic at Children's Hospital of Philadelphia (CHOP) or the Pediatric & Adolescent Care Transgender Services (PACTS) program at Mazzoni Center in Philadelphia (Mazzoni PACTS), who are referred by staff, are invited to attend the support group. At the time of this research, parents and caregivers, transgender children younger than 12, transgender children 13 years of age and older, and cisgender siblings met in separate groups facilitated by professionals. Each group met for ∼90 min, with 15 min before and 30 min after reserved for informal discussion across groups. Parents had the opportunity during their session to discuss challenges they have experienced in the previous month, including issues relating to social and medical transitions, school, family, the political environment, and their relationship with their trans/GNC child. Similarly, the teen group discussed topics of interest to them, while the group for younger children generally involved crafts, games, or stories focused on affirming their identities.

Study participants were recruited through the email listserv and secret Facebook group maintained by and for participating parents, exclusively. To be eligible to participate in the study, subjects were required to (1) be an adult 18 years of age or older; (2) be the biological parent, step parent, foster parent, legal guardian, or parent through adoption of a child 3–22 years of age, who identifies as transgender or gender nonconforming; and (3) have attended one or more sessions of the monthly CHOP-Mazzoni PACTS support group. More than one parent per household was permitted to participate. Surveys were self-administered online through REDCap. As part of the online consent process, parents were told that the survey was for a research study being led by one of the parents in the support group and that all response would be kept confidential. Only if they agreed to participate by checking “yes” were they able to access the survey questions. See Supplementary Appendix S1 for list of survey questions.

This study was approved by the University of Pennsylvania Institutional Review Board.

### Quantitative analysis

Descriptive statistics for close-ended survey responses were computed using SPSS v. 25.

### Qualitative analysis

We used thematic analysis, a method for identifying patterned responses that capture something important about a research question, but cannot be quantified, to analyze the close-ended survey responses.^22^ This involved both authors independently reviewing the closed-ended responses, row by row, in the original spreadsheet format to identify possible themes for further exploration. Together, we reviewed the list of possible themes and organized them around four umbrella (or “parent”) codes: knowledge, social support, limitations, and language used by parents. Both authors then coded all of the close-ended responses, line by line, using Dedoose, a web-based platform for qualitative and mixed-methods data analysis developed by scholars at UCLA. Multiple codes were applied to the same phrases where appropriate. The authors then met to discuss differences in coding and then recoded all of the transcripts a second time, addressing the discrepancies. Both authors then reviewed the final coded material and agreed to divide materials coded under “social support” into “emotional support” and “knowledge” for the purposes of organizing the results.

## Results

### Demographics

The majority of participants were biological parents and female identified. The mean age of their trans/GNC child was 13.9 years (standard deviation 5.1, range 5–22 years), the majority of whom were enrolled in public schools. Their trans/GNC child was more likely to be assigned female at birth and more likely to describe their true gender identity as male (Table 1).

**Table 1.** Demographics of Study Participants

Demographics	Total sample (*N*=48)
Participant relationship to trans/GNC child, *n* (%)	
Biological parent^a^	40 (83.3)
Step parent	1 (2.1)
Adoptive parent	5 (10.4)
Legal guardian	1 (2.1)
Sex of participant, *n* (%)	
Female	37 (77.1)
Male	7 (14.6)
Intersex	1 (2.1)
Gender of participant, *n* (%)	
Female	39 (81.3)
Male	6 (12.5)
Genderfluid or genderqueer	3 (6.3)
Mean age of trans/GNC child (SD, range), *n* (%)	13.9 (5.1)
5–6 Years old	6 (12.5)
7–9 Years old	6 (12.5)
10–12 Years old	7 (14.6)
13–15 Years old	8 (16.7)
16–18 Years old	10 (20.8)
18–22 Years old	9 (18.8)
Trans/GNC child school status, *n* (%)	
Elementary school	16 (33.3)
Middle school	5 (10.4)
High school	15 (31.3)
College	8 (16.7)
Home schooled/online school	4 (8.3)
Type of school for trans/GNC child, *n* (%)	
Public	29 (60.4)
Private	7 (14.6)
Charter	2 (4.2)
Cyber/online	2 (4.2)
Home school	2 (4.2)
Sex assigned at birth for trans/GNC child, *n* (%)	
Female	28 (58.3)
Male	20 (41.7)
Trans/GNC child's true gender identity, *n* (%)	
Female	20 (41.7)
Male	25 (52.1)
Genderfluid or nonbinary	3 (6.3)

^a^ Percentages for some variables may not add up to 100 due to missing data and rounding.

SD, standard deviation; trans/GNC, transgender and gender nonconforming.

### Support group participation

The majority of participants reported attending the support group with their trans/GNC child (70.8%), while fewer reported attending with a spouse (37.5%) or other younger (20.8%) or older children (8.3). Based on the close-ended responses, most reported that attending group was either important or critically important to them (72.9%) and to their trans/GNC child (66.7%). Only 6.3% of parents reported that attending was not particularly important to them, and 10.4% reported that it was not particularly important to their trans/GNC child (Table 2).

**Table 2.** Importance of Support Group to Family Members

Survey question	Total sample (*N*=48), *n* (%)
How important would you say participation in the support group is to you?	
Critical—one of the single most important things I do for support	10 (20.8)
Important—one of several forms of support I have	25 (52.1)
Helpful, but not essential	10 (20.8)
Not particularly important	3 (6.3)
How important would you say participation in the support group is to your Trans/GNC child?	
Critical—one of the single most important things I do for support	17 (35.4)
Important—one of several forms of support I have	15 (31.3)
Helpful, but not essential	11 (22.9)
Not particularly important	5 (10.4)
For whom do you attend the support group? Please choose all that apply.	
I attend the support group because it is important to me	40 (83.3)
I attend the support group because it is important to my spouse/partner	15 (31.3)
I attend the support group because it is important to my trans/GNC child	36 (75.0)
I attend the support group because it is important to my other child(ren)	5 (10.4)
With whom do you usually attend group? Please choose all that apply.	
Spouse	18 (37.5)
Trans/GNC child	34 (70.8)
Older siblings of trans/GNC child	4 (8.3)
Younger siblings of trans/GNC child	10 (20.8)

^a^ Percentages for some variables may not add up to 100 due to rounding.

### Emotional support

Emotional support was the most frequently cited impact for participants, their trans/GNC child, and their family within the close-ended survey results. Participants explained that having a chance to listen to others and share their own experiences helped them to feel less anxious, fearful, and alone.

I love group because we laugh together, we cry together. … It helps us all to see that loving our children happens in lots of different ways and that loving ourselves is critical to this journey.

It is very helpful knowing that you are not alone and there are others who have similar stories. Asking other parents how to proceed with things and just share your feelings is very helpful too.

Listening to other people share their experiences makes me feel better. Being able to tell someone they are not alone in this journey is cathartic to everyone in the room.

I was so grateful to know that I was not alone and others felt the same way as me. It helped to hear all the struggles and the positive outcomes.

It was helpful to be around other parents who understood the anxiety, panic and pain of parenting a trans/nonconforming child. It was a catharsis for everything that I've had to stuff down and pretend to be brave.

I love group because it means I have a cohort of parents who are struggling with similar issues. … it's reassuring to know that other people have the same/similar struggles, particularly around mental health.

Participants described the other parents in the support group as friends, “chosen family,” peers, and part of their support network. These relationships were especially important, given alienation from other family and friends over their decision to support their trans/GNC child. Because they understood the experience of parenting a trans/GNC child, these other parents could provide support without judgment.

The single biggest help to us parents is being in the company of others who do not question the reality of our experience.

I think that I get reaffirmed in my decision to be 100% supportive. I do not always get that outside the group.

Knowing there was a space we could let our guard down. Knowing we wouldn't be judged because of the decisions we made as parents to listen to what our child was telling us.

I love the people I've met at group. Folks there are my chosen family. I know that when I say something either in group or out, these parents will understand what I mean, without having to explain it all or feel like I'm something of an interest or fascination to others.

It has helped balance out all the negative feedback from some family members and it has been helpful to know that you are not alone.

I lost almost all my friends due to my choice to support my child. Group has connected me with other parents and I have made some really close new friends.

Participants also referenced feeling good about being able to offer support to other parents, particularly those newer to the experience of parenting a trans/GNC child.

Sharing with other parents, identifying with the challenges and struggles and realizing that we are in a place to offer support to parents who are new to the trans world … if feels great to empathize and offer affirmation, it is invaluable.

It allows me to give back by aiming to mentor and offer to support other parents who are earlier in their journey with their child.

I come for the friendship and camaraderie and try to give back and mentor those a little earlier on in their journey.

Participants also described the emotional support their trans/GNC child received by attending the support group. Similar to their own perspective, they reported that their children appreciated being in a space with others who could understand their experience.

It's amazing for my daughter to grow up with a whole cohort of other kids who are trans, to see that there are people like her and there are trans/GNC people who aren't exactly like her … She associates being trans as something special and going to group as a treat.

For our daughter, the best part is being social without fear.

It is really the only time when he is in a room full of his peers.

This is the only environment where my child is surrounded by people who understand the overwhelming challenges of transitioning.

The impact for my child was knowing there were other kids like her and also having a space where she wouldn't be judged.

She has difficulties feeling comfortable in social situations, however being around other trans kids seemed to help her … She's felt alone, with no one (but family) to talk to.

They also developed friendships, sometimes the only or the most important friendships for their child.

It's also provided my child with a tribe and a fun Saturday experience.

My child has found some friends. He is very isolated outside the group … he needs this as a respite so he can keep going.

It has impacted my child by providing him a social group of friends with whom he can truly relate to—they have become the best friends he has in his life.

Participants also reported noticing positive differences in the disposition of their trans/GNC child after participating.

As we started to attend the group and also receive services from the clinic, I started to see my daughter appear to have some hope.

It was helpful for my trans child, she seemed so much lighter and excited. She never really gets excited about much anymore.

My child is definitely more vocal about what he wants for himself. Our relationship has bettered because we can now ‘breathe’ and talk about anything related to this.

Participants also referenced that participation in the support group provided emotional support for their whole family.

It is the only place my husband and daughters and myself feel like we have found others who truly understand this journey.

It was also so important for her sibling. He really needed to be around other kids who are trying to navigate the changes happening. He's felt pretty isolated since his sister came out to the family but not classmates.

## Knowledge

In addition to emotional support, parents described in the close-ended responses how they gained knowledge about the medical, legal, and political aspects of parenting a trans/GNC child through participation in the support group. Hearing about the experiences of other parents, including parents with older children and those further along in their transition, helped parents make sense, intellectually, of the choices they faced and to navigate complex issues.

There is a huge learning curve in understanding the needs of a transgender child … We did not have the knowledge and understanding to help our child properly and had to do much work to get to where we are now. We, as college-educated parents with modest income, found that supporting our transgender child was very difficult.

I love that I am able to ask questions regarding other trans kids. Since it's a mixed group of people with children of all ages, I'm able to ask what I may expect in the future, how have other children reacted or coped in certain situations.

Participants gained knowledge regarding issues of medical transition, including puberty blockers, menstrual suppression, cross-hormone use, and top surgery. In addition to learning about the physical health aspects, they shared information with other parents about specific doctors and health insurance coverage. Similarly, participants reported learning from other parents about the process of legally changing their child's name and correcting their gender marker. Participants also learned from fellow parents about how to advocate for their child, particularly in school and medical settings.

### Limitations of support group

The support group was not all things to all participants. Survey responses were overwhelmingly positive, but a relatively small group had a very different experience, including three who responded that the support group was “not particularly important” to them and five who responded that the support group was “not particularly important” to their trans/GNC child. Some of the concerns and negative experiences centered on being overwhelmed and feeling judged.

It was not a positive experience AT ALL. I made recommendations to split the parents by age because it's way too overwhelming to be in the beginning of the process and sit and listen to parents speak about top/bottom surgery. Additionally, I felt attacked by other parents when I didn't use female pronouns for my child—- um hello isn't the journey individual for all included??? I wasn't ready! The SAME parents seemed to ‘run’ the group—- monopolizing the conversation.

Other concerns focused on the size and composition of the group for themselves or their trans/GNC child. Parents lamented that the group had become too large, “overcrowded,” and “less personal.”

I do feel like sometimes people come to group with really weighty issues and very much in need of support and I often wonder if the group is structured to meet those needs as we are so big.

Others indicated that their trans/GNC children were not able to find peers of the same age and gender identity and had trouble connecting with the other children.

Our daughter has not asked to go back since she was with little children and did not have much in common.

Child was reticent, as they didn't have any gender fluid kids their age in the group at the time.

This past meeting was the 4th meeting and none of the children really speak to my son. When we go to the coffee shop afterward, it's hard for me to see my son sitting with the others but not really being a part. I know this bothers him, but he still looks forward to coming for the activities, the adult welcoming and just to be a part. It's better than nothing.

Other participants noted the lack of racial diversity, particularly families of color.

I still yearn the camaraderie of families of color that I can identify more with.

We have very little racial diversity in the support group. We are just starting to see a few families of color participate. I hope the group can meet their needs and that we can start to see more families of color join.

For others, the support group played an important role at one particular time for themselves and their trans/GNC child. As they moved past those moments, they felt they needed the support group less.

I no longer attend, but only because I don't need lots of support now.

Being 3 ½ years along into the journey with our son, I feel like we are through the hardest part of the transition—that first 2 years. We don't face a lot of difficult decisions anymore and our son is in a really good place.

More responses noted the logistical challenges of participating in Saturday morning support group because of work, childcare, and transportation issues. One participant noted that they drove 2 h each way to get to group; another noted that they stay overnight in Philadelphia to attend. Still others noted discomfort driving in the city.

## Discussion

This study points to one specific way—participation in an in-person support group—for parents of trans/GNC children to find the support they need to support their children. The perceived benefit was widespread; three-quarters of parents reported that they found the group either critical or important to themselves and two-thirds of them said it was critical or important to their trans/GNC child. Our findings are consistent with those from studies on the participation of parents of children with special emotional, developmental, and cognitive needs in support groups. Being with other parents who were experiencing the same or similar challenges—”perceived sameness”—provided unique emotional support and opportunities for developing knowledge and skills. Participation led to “mutuality of support,” new connections, and “equitable” friendships for parents and their trans/GNC children that involved “no judgment.”^18^ Participation helped family dynamics, bringing joy and hope back to some trans/GNC children and opening up channels of communication within families.^18^ Perhaps unconsciously, parents and caregivers developed common language and a shared narrative around their experience of parenting a trans/GNC child, anchored by the sentiment that they were supporting their trans/GNC child. This helped to normalize their experience and offered some protection from family, friends, and a society who would blame them for the challenges they faced.^17^

The use of a common language emerged as a theme in survey responses even though participants did not make reference to it; they may or may not be aware that one byproduct of participating in the support group was development of a shared language. The repeated use of words such as “journey,” “coming out,” “transition,” “assigned sex,” “gender identity,” “presenting male/female,” and “gender confirmation surgery” suggest not just a common language, but a common understanding, even a common narrative, about their parenting experiences, consistent with the findings of Frigerio and Montali that development of a “shared narrative” helps absolve parents of guilt and maintain a sense of being “good parents.”

## Limitations

This study has several limitations. As participants in the support group, ourselves, we were in a position to report on the experiences of other parents, but not objectively assess the impact participation on their level of acceptance for their trans/GNC child. Participation in the CHOP-Mazzoni PACTS support group is restricted to families that are receiving specialized medical care and determined by professionals to be likely to benefit from participation in a support group. In other words, they are parents and caregivers who have already demonstrated some level of acceptance for their trans/GNC child. It is not clear if parents who are less accepting or whose children are not receiving specialty gender health care would benefit in the same way, if at all. The CHOP-Mazzoni PACTS model involves professional staff to facilitate each of the separate groups. It is not clear what impact participation in support groups organized exclusively by parent peers would have. Participants in the CHOP-Mazzoni PACTS support group are overwhelmingly white and have private health insurance, so much so that we did not ask race/ethnicity, income, or insurance status on the survey because it might have allowed us to link survey responses to individual families. Would people of color experience the support group similarly? Would low-income families receive the same perceived benefits from participation?

Our participants were mostly female; only 7 out of 48 participants identified as male. Do fathers and other male-identified adults in the lives of trans/GNC children experience participation in a support group the same way? We did not ask about marital status in the survey. How does the experience of two-parent households compare with single-parent or divorced households? How do blended families navigate participation in the support group?

The fact that participants used a common language in describing their experiences in parenting a trans/GNC child may have been influenced, in part, by the wording of the open-ended questions. We included the phrases “sex assigned at birth,” “true gender,” “journey,” “come out,” and “social, legal, or medical transition.” Of course, our own use of this language in the wording of the survey may serve as additional evidence of the impact of participating in the support group. Survey participants knew the results would be analyzed by fellow parents in the support group. On the one hand, this may have motivated them to complete the survey, but it may also have influenced how they answered.

## Conclusion

Additional research focused on all of these topics—the role and experience of fathers, the experience of single-parent and divorced households, the value of support groups for families of color and families without private health insurance, and the development and utility of a shared narrative—would all be valuable. Furthermore, additional research should consider the role that professional counseling, participation in online support groups, and other practices provide parents and caregivers with the support they need to support their children and the relationship between these types of support and support group participation.

While the generalizability of our results to broader groups of parents of trans/GNC children is not clear, our findings provide evidence that support groups may offer a scaleable and cost-effective approach to addressing the common fears, isolation, and confusion that parents of trans/GNC children face. This study underscores the importance of parents' voices—as members of family system negotiating the gender transition of a child, as participants in a support group, and as researchers—to the growing discussion about how best to support trans/GNC children and their families.

## Supplementary Material

Supplemental data

## Abbreviations Used

CHOP: Children's Hospital of Philadelphia
PACTS: Pediatric & Adolescent Care Transgender Services
trans/GNC: transgender and gender nonconforming
