# Supplementary material for: Parent Participation in a Support Group for Families with Transgender and Gender-Nonconforming Children: “Being in the Company of Others Who Do Not Question the Reality of Our Experience”
Source: Transgend Health. 2019 Aug 12;4(1):168–75. doi: 10.1089/trgh.2018.0018 (PMC6689185; doi:10.1089/trgh.2018.0018)
Supplement: Supplemental data [file Supp_AppendixS1.pdf]

Supplementary Data

SUPPLEMENTARY APPENDIX S1. Survey of Parents of Transgender and Gender-Nonconforming Children

This survey is being conducted by [redacted], an Associate Professor at the University of Pennsylvania's School of Social Policy & Practice. You are being asked to participate because you are a member of the CHOP-Mazzoni Support Group for families with transgender and gender-nonconforming children. This survey aims to assess the experiences of parents with the support group and other forms of peer support from fellow parents of trans/GNC children. Participation in this survey is entirely voluntary. There are no risks or direct benefits to you for participating. You will not be asked to share your name, your child's name, or your contact information in this survey. All information you provide will be kept confidential.

Adults 18 years of age and older, who are the biological parent, step parent, foster parent, legal guardian, or parent through adoption of a child ages 3–22, who identifies as transgender or gender nonconforming (this includes gender fluid, gender nonbinary, agender, and gender queer), and who has attended one or more sessions of the monthly CHOP-Mazzoni support group are invited to participate. This survey is not intended for other parents of trans/GNC children, who you may know through other personal or online communities.

Do you agree to participate? *(yes/no)*

> If you have questions about this survey, you may contact [redacted]. If you have any concern about this research study, you may contact the Office of Regulatory Affairs at the University of Pennsylvania by calling (215) 898–2614.

PART I: This section is intended to provide background on you and your family. You will be invited to offer open-ended responses in Part II.

1. Are you the parent or guardian of a transgender or gender-nonconforming child age 3–22?

   Yes
   No

2. Please describe your relationship to that child:

   Biological parent
   Step parent
   Parent by adoption
   Foster parent
   Legal Guardian

3. Please describe your gender

   Male
   Female
   Something else not listed here

4. How old is your child? If you have more than one trans/GNC child, please answer these initial questions based on the youngest child.

   3–5 years old
   6–10 years old
   11–14 years old
   15–18 years old
   19–22 years old

5. Is your child currently enrolled in school?

   Yes
   No

6. If enrolled in school, what grade level?

   Preschool
   Elementary school
   Middle school
   High school
   College
   Graduate school

7. If enrolled in school, what type of school?

   Public
   Private
   Charter
   Parochial/religious

8. In what state do you live?

   NJ
   PA
   State not listed

9. Who lives in the home with your child?

   You (parent/guardian completing the survey)
   Your spouse/partner
   Older sibling(s)
   Younger sibling(s)
   Twin sibling
   Other relatives
   Friends

10. What sex was your child assigned at birth?

    Male
    Female
    Intersex

11. How important is participation in the support group to you?

    Critical—one of the single most important things I do for support
    Important—one of several forms of support I have
    Helpful, but not essential
    Not particularly important

12. How important is participation in the support group to your nonbinary/trans child?

    Critical—one of the single most important things they do for support
    Important—one of several forms of support they have
    Helpful, but not essential
    Not particularly important

Part II. In this second section, please answer the questions as completely as you can. Please avoid using names of people (your children and family, health care providers, etc) or institutions (such as your child's school or health care agency) to protect confidentiality.

13. Where is your child and your family in the transition process? In other words, when did your child come out as nonbinary/transgender and how far along is your child in their social, legal, and medical transition? At what point in the transition did you join the support group?

14. Please describe your experience participating in the CHOP-Mazzoni support group. In what specific ways has it been helpful to you?

15. How has participation in support group impacted your decisions and choices about how to support and seek care for your child?

16. How has participation in the support group impacted your role as parent/guardian of a trans child? How has participation in the support group impacted on your child?

17. In what other ways do you communicate with parents in the CHOP-Mazzoni support group? This might include face-to-face outside the support group, text, email, phone calls, Facebook, etc. Which of these is most helpful?

18. Please indicate anything else you would like to share with us, about your journey with your child or feedback about this survey.
